# Cooled radiofrequency ablation provides extended clinical utility in the management of knee osteoarthritis: 12-month results from a prospective, multi-center, randomized, cross-over trial comparing cooled radiofrequency ablation to a single hyaluronic acid injection

**DOI:** 10.1186/s12891-020-03380-5

**Published:** 2020-06-09

**Authors:** Antonia F. Chen, Fred Khalouf, Keith Zora, Michael DePalma, Lynn Kohan, Maged Guirguis, Douglas Beall, Eric Loudermilk, Matthew J. Pingree, Ignacio Badiola, Jeffrey Lyman

**Affiliations:** 1 Department of Orthopaedics, Brigham and Women’s Hospital, 75 Francis St, Boston, MA 02115 USA; 2 University Orthopedics Center, 3000 Fairway Dr, Altoona, PA 16602 USA; 3 University Orthopedics Center, 476 Rolling Ridge Drive, State College, PA 16801 USA; 4 Virginia iSpine Physicians, 9020 Stony Point Pkwy #140, Richmond, VA 23235 USA; 5 University of Virginia School of Medicine, 545 Ray C Hunt Drive, Charlottesville, VA 22903 USA; 6 Ochsner Clinic Foundation, 2820 Napoleon Ave, Ste 210A, New Orleans, LA 70115 USA; 7 Clinical Investigations, 1800 Renaissance Blvd Suite 110, Edmond, OK 73013 USA; 8 PCPMG Clinical Research Unit LLC, 100 Healthy Way #1260, Anderson, SC 29621 USA; 9 Mayo Clinic, 200 1st St SW, Rochester, MN 55905 USA; 10 University of Pennsylvania, 3737 Market Street Room 6113, Philadelphia, PA 19104 USA; 11 Institute for Orthopedic Research and Innovation, 1110 W Park Place, Suite 212, Coeur d’Alene, ID 83814 USA

**Keywords:** Osteoarthritis, Denervation, Radiofrequency ablation, Non-surgical

## Abstract

**Background:**

Safe and effective non-surgical treatments are an important part of the knee osteoarthritis (OA) treatment algorithm. Cooled radiofrequency ablation (CRFA) and hyaluronic acid (HA) injections are two commonly used modalities to manage symptoms associated with knee OA.

**Methods:**

A prospective 1:1 randomized study was conducted in 177 patients comparing CRFA to HA injection with follow-ups at 1, 3, 6 and 12 months. HA subjects with unsatisfactory outcomes at 6-months were allowed to crossover and receive CRFA. Knee pain (numeric rating scale = NRS), WOMAC Index (pain, stiffness and physical function), overall quality of life (global perceived effect = GPE, EQ-5D-5 L), and adverse events were measured.

**Results:**

At 12-months, 65.2% of subjects in the CRFA cohort reported ≥50% pain relief from baseline. Mean NRS pain score was 2.8 ± 2.4 at 12 months (baseline 6.9 ± 0.8). Subjects in the CRFA cohort saw a 46.2% improvement in total WOMAC score at the 12-month timepoint. 64.5% of subjects in the crossover cohort reported ≥50% pain relief from baseline, with a mean NRS pain score of 3.0 ± 2.4 at 12 months (baseline 7.0 ± 1.0). After receiving CRFA, subjects in the crossover cohort had a 27.5% improvement in total WOMAC score. All subjects receiving CRFA reported significant improvement in quality of life. There were no serious adverse events related to either procedure and overall adverse event profiles were similar.

**Conclusion:**

A majority of subjects treated with CRFA demonstrated sustained knee pain relief for at least 12-months. Additionally, CRFA provided significant pain relief for HA subjects who crossed over 6 months after treatment.

**Trial registration:**

This trial was registered on ClinicalTrials.gov, NCT03381248. Registered 27 December 2017

## Background

Knee osteoarthritis (OA) is a painful and debilitating disease that often affects patients for years [1]. While total knee arthroplasty (TKA) is widely considered a definitive treatment for late stage knee OA, non-surgical options are useful for symptomatic management. Patients experiencing knee OA suffer from pain an average of 9 years before becoming candidates for surgical intervention [2].

Nonsurgical treatment options for knee OA symptoms include weight loss, activity modification and physical therapy [3]. If these do not provide adequate relief, nonsteroidal anti-inflammatory drugs (NSAIDs) and acetaminophen can be taken to mitigate pain. However, it should be noted that these pharmacologic interventions can present significant adverse events (AEs) [4]. Intra-articular steroids (IAS) injections have been utilized to manage knee OA symptoms, but studies have demonstrated they may only provide short-term pain relief [5, 6]. Other studies have cautioned that multiple steroid injections may lead to accelerated knee osteoarthritis progression [7]. Platelet rich plasma injections have also been employed to manage knee OA pain, but questions remain regarding efficacy and the lack of standardization of treatments [8–10]. Hyaluronic acid (HA), otherwise known as “viscosupplementation” injections, are another treatment option. Some clinical trials have shown modest effects of HA injections when managing knee OA pain [11], but larger statistical analysis have concluded the benefits of HA are clinically insignificant [12].

Radiofrequency ablation (RFA) is the targeted delivery of radiofrequency energy through a probe that causes the thermal degradation of nerve structures via ionic heating. The areas of thermal degradation are referred to as lesions. Traditional radiofrequency ablation probes operate at a set temperature of 80 °C. Cooled radiofrequency ablation (CRFA) uses internally cooled radiofrequency probes which are able to delivery more energy to surrounding tissues. While internally cooled probes operate at a set temperature of 60 °C, temperatures in tissues beyond the probe tip reach 80 °C. As a result of the internal cooling of the probe, larger lesions are created which can help overcome physiological variability of nerve location and increase the likelihood of treatment success [13]. Additionally, recent research has highlighted distinct physiological differences between lesions created by CRFA probes compared to standard radiofrequency probes, that may account for the extended durability of pain relief when using CRFA [14]. Clinical studies have demonstrated that CRFA can provide 12-months of pain relief for the majority of patients undergoing this procedure [15–17]. A subset of subjects receiving CRFA have reported pain relief extending through the 18 and 24-month timepoints [18]. A previous study demonstrated the effectiveness of CRFA versus IAS injections [15, 18, 19] and CRFA vs HA at 6-months [20], but no study has compared CRFA to HA with 12-month follow-up and a cohort of HA patients that could crossover to receive CRFA after 6-months.

Thus, the purposes of this study were to: (1) evaluate the efficacy of CRFA for the treatment of knee OA pain at 12 months, and (2) evaluate HA patients who crossed over to CRFA after 6-months of treatment.

## Methods

### Study design

This prospective, randomized, multi-center study was originally designed to compare the extent of OA-related knee pain relief in subjects receiving CRFA (COOLIEF*, Avanos Medical, Alpharetta, GA, USA) of genicular nerves or a single intra-articular HA injection (Synvisc-One® (Hylan G-F 20); Sanofi, Bridgewater, NJ, USA). Subjects were randomized in a 1:1 randomization scheme, with post-treatment data collection occurring at 1, 3, 6 and 12 months. This study adheres to CONSORT guidelines and a flow-diagram is reported in the results section.

Per study protocol, subjects initially randomized to the HA cohort who were deemed medically appropriate for CRFA at 6 months were eligible to crossover. Medical appropriateness was determined by each investigator by factoring in both safety considerations and patient preferences. Subjects did not need to formally requalify for the study to receive crossover per the inclusion/exclusion criteria; however, confirmation and documentation was needed that subjects remain medically appropriate candidates for the CRFA procedure in order to be eligible. This was a single-arm crossover trial; subjects originally receiving CRFA were not given the option to crossover to receive HA. Subjects within the HA cohort that did not crossover were followed. Follow-up for all cohorts (CRFA, crossover and HA) occurred at 12-months.

### Study subjects

All subjects that presented with signs and symptoms of knee OA were considered for the trial. Full descriptions of primary inclusion study criteria are shown in Table 1.

**Table 1:** Inclusion Criteria (NRS = Numeric Rating Scale; MRI = magnetic resonance imaging, CT = computed tomography, OA = osteoarthritis)

Age ≥ 21 years.	
Able to understand the informed consent form and provide written informed consent and able to complete outcome measures.	
Chronic knee pain for longer than 6 months that interferes with functional activities	
Continued pain in the target knee despite at least 3 months of conservative treatments	
Positive response (defined as a decrease in numeric pain scores of at least 50%) to a single genicular nerve block of the index knee.	
Pain on NRS ≥ 6 on an 11-point scale for the index knee.	
Radiologic confirmation of arthritis (x-ray/MRI/CT) of OA grade of 2 (mild), 3 (moderate) or 4 (severe) noted within 6 months for the index knee.	

Diagnosis of knee OA for each trial candidate was determined according to medical history, presentation, physical exam and radiologic confirmation of Kellgren-Lawrence OA grades 2, 3 or 4 [21].

### Diagnostic block and randomization

Subjects meeting inclusion criteria received a diagnostic block of each target genicular nerve according to previously published procedures [19, 22]. Diagnostic blocks consisted of fluoroscopy-guided injections with a small volume (0.60–0.75 mL at each site) of short-acting local anesthetics (preferably Marcaine 0.5% or similar). Diagnostic blocks are part of the treatment algorithm for CRFA and are often required as part of ensuring coverage for the procedure. While diagnostic blocks have been shown to provide pain relief up to two weeks [23], the mean time between block and procedure was 13.1 ± 10.1 days. Baseline pain scores were reported prior to receiving blocks and the first timepoint post-procedure was 30 days, suggesting that reductions in pain were well beyond the two-week window of diagnostic blocks effects and therefor not influenced by diagnostic block Subjects experiencing a ≥ 50% decrease in pain score, as measured by Numeric Rating Scale (NRS), within 15 min were deemed positive responders and were subsequently randomized to their respective cohorts. Subjects randomized to receive CRFA had a mean reduction in pain of 91.3% ± 13.7. Subjects randomized to receive HA had a mean reduction of pain of 92.5% ± 12.6. Those randomized to the HA cohort that elected to crossover were not required to undergo a second round of diagnostic blocks, as they had already responded to diagnostic block prior to randomization.

### Cooled radiofrequency ablation (CRFA cohort)

Subjects randomized to CRFA underwent genicular ablation similar to previously published methods [15, 19].

### Intra-articular hyaluronic acid injection (HA cohort)

Subjects randomized to HA received a single, 6 mL intra-articular dose in accordance with the Instructions for Use (IFU). While there are many hyaluronic acid injectable products, Synvisc-One® was selected because, at the time of trial inception, it was the most commonly used product. Those within this cohort that did not elect to crossover at 6 months were followed to the 12-month timepoint and continued to report their outcomes.

### 6 month crossover cohort

Subjects within the HA cohort who were deemed medically appropriate at the 6-month timepoint were allowed to crossover and receive CRFA treatment. Those within the CRFA cohort were not presented the option to crossover as part of the single-arm crossover design of the study.

### Study outcomes

Knee pain was measured by the 11-point NRS (score of 0 = no pain; 10 = worst pain) at all timepoints [24]. The percentage of subjects reporting ≥50% pain relief following treatment was recorded for CRFA and crossover cohorts. Knee pain, function and stiffness were measured by the Western Ontario & McMaster University Osteoarthritis Index (WOMAC) [25]. Subject’s perception of treatment effectiveness was measured by global perceived effect (GPE) [26] scale and the EQ-5D-5 L Health Related Quality of Life Questionnaire [27]. The GPE scale is a 7-question scale that asks subjects to rate their condition after receiving treatment, where 1 = worst ever and 7 = best ever. The EQ-5D-5 L questionnaire asks patients to rank their overall health status from 0.0–1.0, where 0.0 = worse than death and 1.0 = state of perfect health. All endpoints were measured at baseline (except GPE) and the 1, 3, 6 and 12-month timepoints. Subjects were evaluated for AEs and serious AEs (SAEs) at each visit. Subjects reporting for 12-month follow-up underwent x-ray imaging to monitor for any progression of OA severity. Medication usage was tracked with subjects divided into narcotic analgesics (measured by morphine equivalents) as well as non-narcotic analgesic medications. As this manuscript is the 12-month follow-up to the previously reported 6-month outcomes [20], this extension was not powered for intra-group comparisons. However, the same endpoints were assessed in all cohorts (CRFA and crossover).

### Data analysis

Data management, study site monitoring, and statistics services were performed by a third party independent of Avanos Medical. The original 6-month study had a non-inferiority approach on response rate that was used to estimate the sample size for this study, with “response” defined as ≥50% reduction in pain on the NRS from baseline. The study was not powered to show a difference between the CRFA and HA groups at the 12-month follow-up timepoint, although effectiveness measures (NRS, WOMAC, GPE, EQ-5D-5 L) had pre-specified hypotheses at the 12-month timepoint. A fixed sequence testing procedure was implemented to control family-wise error rate. Continuous data are reported as mean ± standard deviation (range minimum - maximum). Categorical data is summarized as percentages.

### Recruiting and data collection

Recruitment for the trial began 12/4/17. Data presented within this manuscript was collected from 12/7/17 until 8/1/19.

## Results

### Disposition of study subjects

**Fig. 1:**
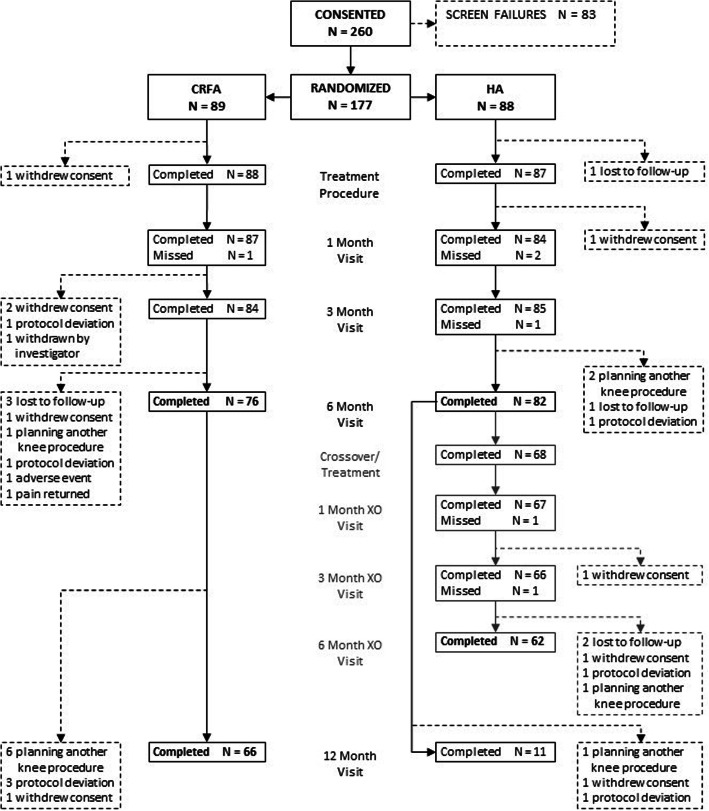
CONSORT Diagram (XO = crossover)

A total of 260 subjects were consented. 177 subjects proceeded to randomization (*n* = 89 CRFA, *n* = 88 HA). 76 subjects in the CRFA cohort completed their 6-month follow-up. A total of 66 subjects within this cohort reported 12-month outcomes. Of the 88 subjects randomized to the HA cohort, 82 completed the 6-month follow-up. Of these, 68 (82.9%) elected to crossover and receive CRFA treatment. 62 of these subjects returned for their 6-month crossover follow-up. 14 subjects in the original HA cohort did not elect to crossover and 11 completed their 12-month follow-up (Fig. 1).

### Demographics

Demographic characteristics between the initial cohorts (CRFA and HA) were similar [20]. Per the original demographic analysis, subjects in the CRFA and HA cohorts were equivalent, with no statistically significant differences observed between cohorts with respect to mean age at consent, mean duration of OA knee pain and gender or ethnicity (*p* > 0.05). Mean body mass index (BMI) was significantly higher in the CRFA group. In both cohorts, the majority of subjects had OA grades 3 and 4 (*p* = 0.2001). Demographic characteristics between the CRFA and crossover cohort were also similar, with no statistically significant differences except for BMI, which was higher in the CRFA group (Additional file 1 Table 1). As the crossover cohort consisted entirely of those in the initial HA cohort, this result was not surprising.

### Knee pain

**Fig. 2:**
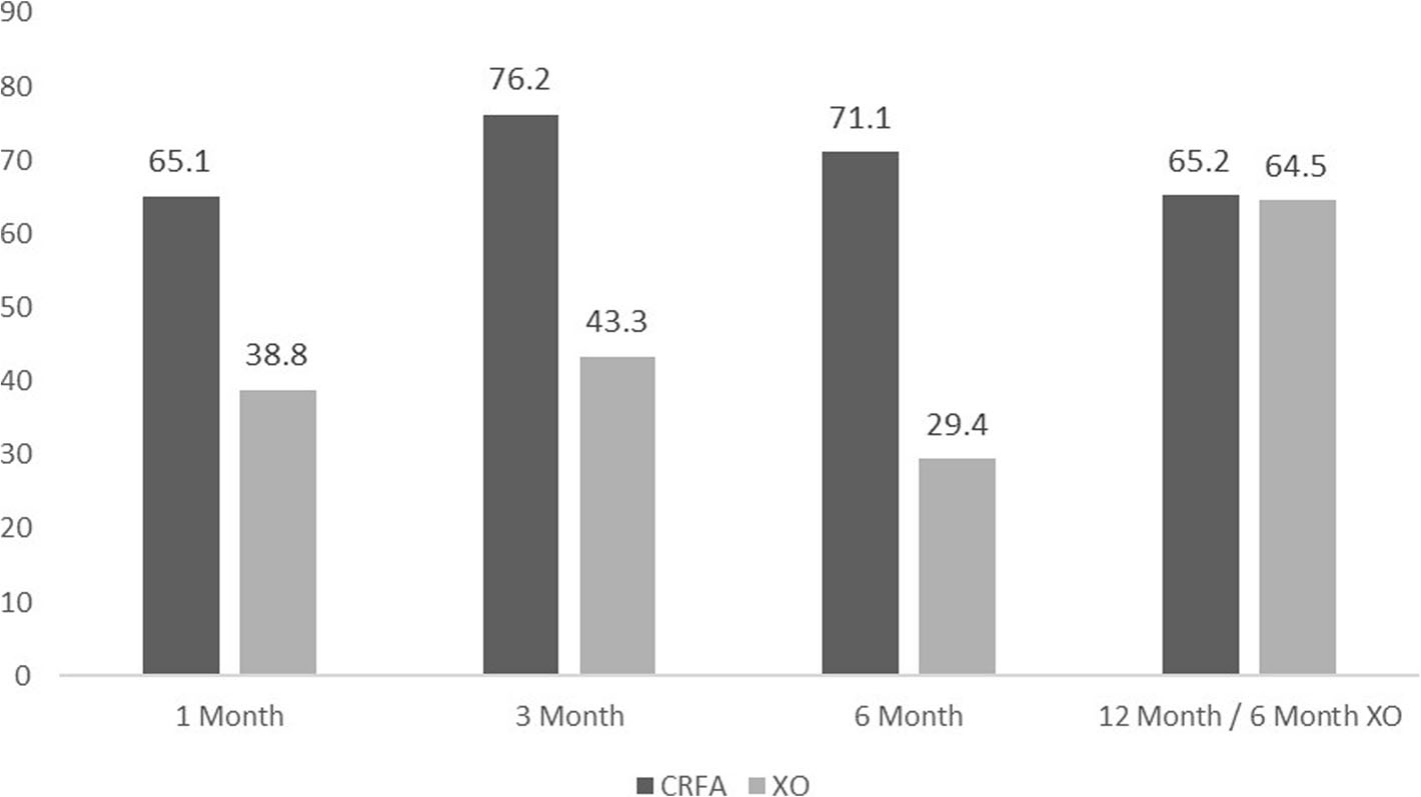
Percent of Subjects Reporting ≥50% Pain Relief (CRFA = cooled radiofrequency ablation, XO = crossover)

At 12 months, 43 out of 66 subjects (65.2%) of the original CRFA group had pain reduction ≥50%, as measured by NRS (Fig. 2). During the original 6-month post-treatment interval (i.e. 1, 3, and 6-month timepoints following HA injection), those within the crossover cohort reported diminishing pain relief, with only 20 out of 68 (29.4%) reporting ≥50% relief at 6-months. However, upon crossing-over, the crossover cohort saw improvements in pain relief, with 40 out of 62 (64.5%) of subjects reporting ≥50% relief at 12-month follow-up or the 6-month crossover timepoint (defined as 6-months after receiving CRFA) compared to their baseline pain, measured at the 6-month timepoint post HA injection but prior to crossing over.

**Fig. 3:**
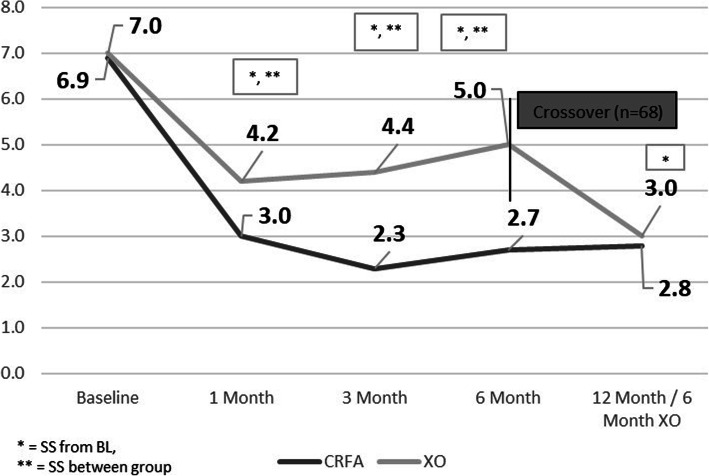
Numeric Rating Scale Pain Scores (SS = statistically significant, BL = baseline, CRFA = cooled radiofrequency ablation, XO = crossover)

Of those originally treated with CRFA, NRS pain scores decreased significantly at all timepoints and maintained pain relief through the 12-month timepoint. At 12-months, the mean NRS pain score was 2.8 ± 2.4 (range 0.0–9.0), compared to a baseline of 6.9 ± 0.8 (range 6.0–9.0), representing a 4.1 decrease in NRS pain score (*p* < 0.0001). Those within the crossover cohort saw an initial decrease in NRS pain score at 1 month after HA treatment, but this score steadily increased at the 3 and 6-month timepoints. At the 6-month timepoint, the crossover cohort had a mean NRS score of 5.1 ± 2.5 (range 0.0–0.0). 6 months after receiving CRFA, subjects in the crossover cohort had a mean NRS of 3.0 ± 2.4 (range 0.0–9.0) (Fig. 3; Additional file 2 Table **2**). Subjects within the crossover cohort had a mean decrease in NRS score of 4.0 ± 2.6 (range − 2.0-8.0) from baseline (*p* < 0.0001). This cohort saw a mean reduction in NRS from 6 to 12 months of 2.0 ± 2.5 (range − 2.0-10.0) from baseline, defined as 6-months after HA but prior to receiving CRFA (p < 0.0001).

### General knee condition following study intervention

Subjects in the original CRFA cohort saw durable improvements in total WOMAC score at 12 months, with a mean WOMAC score of 33.2 ± 23.2 (range 0.0–87.5) (p < 0.0001), representing a 46.2% improvement. Of note, subjects within the CRFA cohort also experienced improvements in WOMAC pain score, reporting scores of 31.7 ± 25.6 (range − 30.0-95.0) (p < 0.0001). This was a 46.8% improvement from baseline.

**Fig. 4:**
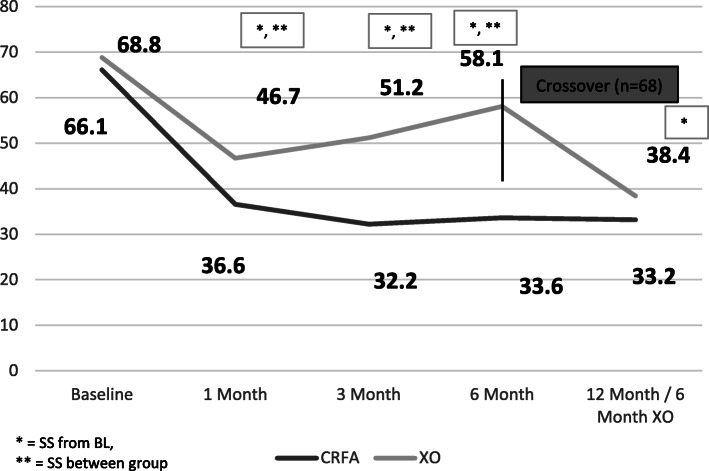
Total Western Ontario & McMaster University Osteoarthritis Index (WOMAC) Score (SS = statistically significant, BL = baseline, CRFA = cooled radiofrequency ablation, XO = crossover)

Within the crossover cohort, after an initial decrease in mean total WOMAC score after HA treatment, there was a steady increase in WOMAC score from the 3 to 6-month period. After crossing over to receive CRFA treatment, those within this cohort had a mean total WOMAC score of 38.4 ± 22.3 (range 0.0–84.4). This was a mean decrease of 18.1 ± 22.1 (range − 20.8-80.2) (*p* < 0.0001). This represented a 27.5% improvement in WOMAC score compared to their adjusted baseline of 6-months post-HA injection (Fig. 4, Additional file 3 Table 3).

### General health of subjects

**Fig. 5:**
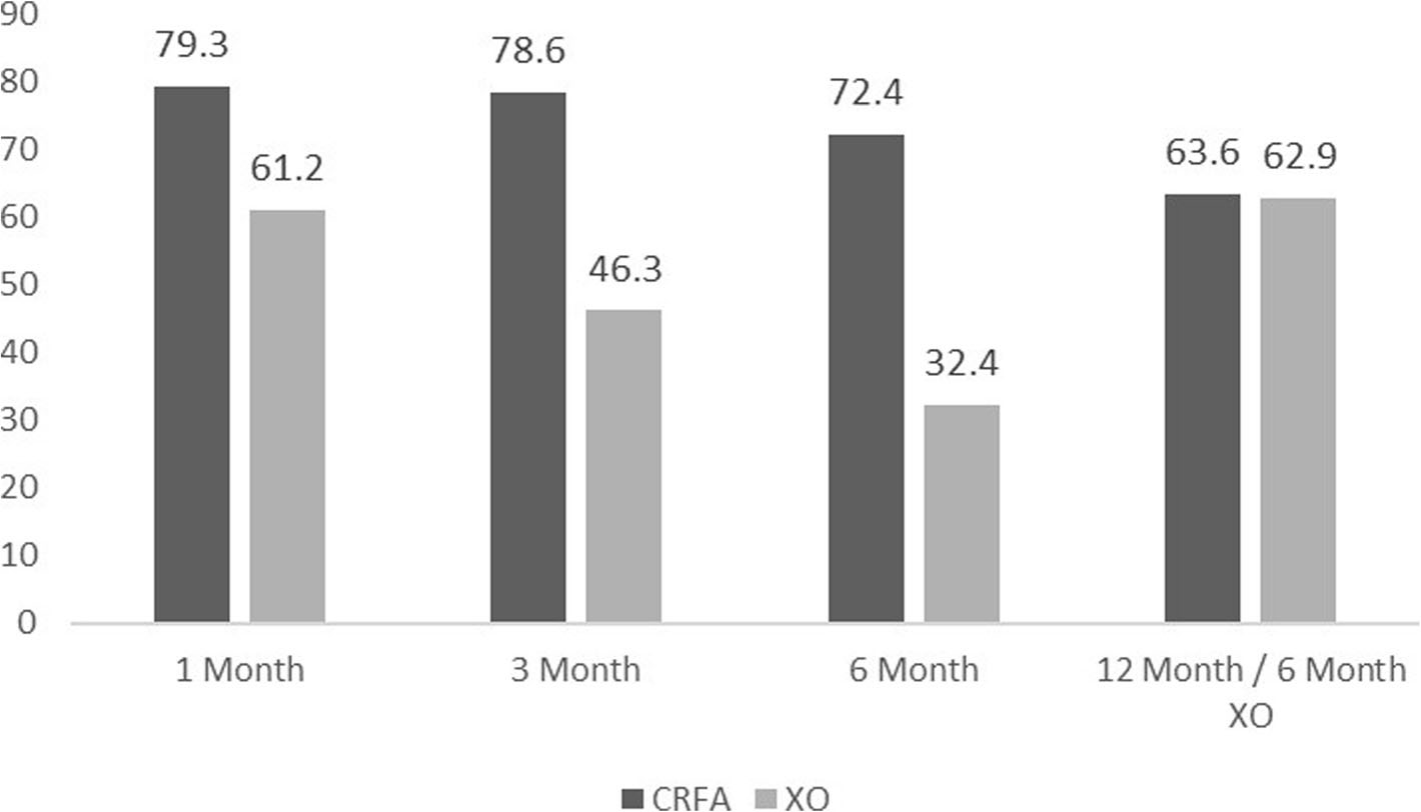
Percent of Subjects Reporting Improved Global Perceived Effect (GPE) (CRFA = cooled radiofrequency ablation, XO = crossover)

In the original CRFA cohort, 63.6% of subjects reported improved knee condition (using GPE) at the 12-month timepoint. In the crossover cohort, there was a downward trend in the rating, with only 32.4% of subjects reporting improved knee condition at the 6-month timepoint after HA injection. After crossing-over, 62.9% of subjects in the crossover cohort reported improved knee condition compared to their adjusted baseline of 6-months post-HA injection (*p* = 0.93) (Fig. 5, Additional file 4 Table 4).

**Fig. 6:**
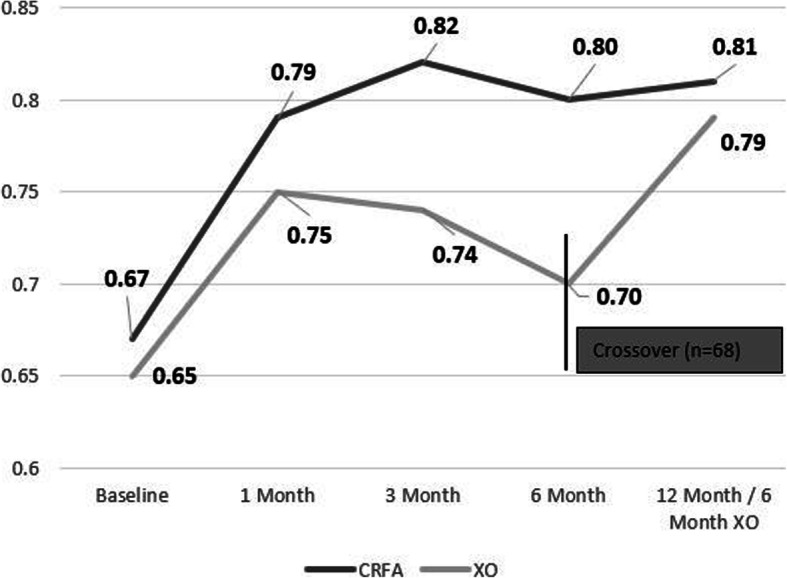
EQ-5D-5 L Scores (CRFA = cooled radiofrequency ablation, XO = crossover)

Subjects within the CRFA cohort had a sustained improvement in general health based on EQ-5D-5 L. At the 12-month timepoint, this cohort had a score of 0.81 ± 0.10 (range 0.58–1.00), compared to a baseline of 0.67, representing a mean change of 0.12 (*p* < 0.0001). Those in the crossover cohort also reported an improved EQ-5D-5 L score of 0.79 ± 0.14 (range 0.35–1.00) at the 12-month timepoint (*p* < 0.0001) (Fig. 6, Additional file 5 Table 5).

### Medication usage

Narcotic usage between all subjects was recorded throughout the trial. At baseline, 8 subjects in the original CRFA cohort and 7 subjects in the crossover cohort reported taking opioids. After 12 months, 6 subjects in the CRFA cohort and 6 in the crossover group reported taking opioids. There were no statistically significant changes in opioid medication usage over time (*p* = 0.6205). However, there was no increase in opioid usage reported between 6 and 12 months in either cohort. Of subjects taking opioid medication at baseline, within the CRFA cohort, 5 were taking opioids for knee pain only, 1 was taking opioids for non-knee related pain, and one was taking opioids for knee and other pain. Within the HA cohort, 7 were taking opioids for knee only, 1 was taking opioids for non-knee pain, 2 were taking opioids for knee and other pain, and 1 was taking opioids to unspecified reasons. Overall, only 12 subjects were taking opioids for knee related pain. Given the small sample size, it was difficult to determine significance in reduction.

At baseline, 47 in the CRFA cohort and 31 in the crossover cohort were taking non-narcotic analgesics. At 12-months, 28 subjects in the CRFA cohort and 26 in the crossover group were taking non-narcotic analgesics. There were no statistically different changes from baseline in either group with non-opioid pain medications (*p* = 0.6539), although within the CRFA group it trended towards reduction (mean reduction of 156.8 mg/day) 12 months after treatment.

### HA cohort results

Of note, 14/87 (16%) subjects in the original HA cohort were not deemed medically appropriate candidates for CRFA and did not elect to crossover. Of the 11 that returned for their 12-month follow-up, 10 (90.9%) reported ≥50% pain relief at the 12-month timepoint. NRS scores for this cohort were 6.9 ± 0.8 (range 6.0–9.0) at baseline (*n* = 20), 1.9 ± 2.1 (range 0.0–6.0) at 6 months (*n* = 14) and 1.5 ± 1.4 (range 0.0–4.0) at 12 months (*n* = 11).

### Radiographic analysis

Radiographic exams were completed at the final visit and across all subjects receiving CRFA, the majority of patients remained the same OA grade as they were upon entry into the trial. In the CRFA cohort, 84.6% (55/65) had no change in OA grade. In the crossover cohort, 64.5% (40/62) had no change in OA grade.

### Subgroup analysis of CRFA responders by Kellgren-Lawrence grade

Subgroup analysis was performed on subjects within the CRFA cohort based on the primary outcome of ≥50% pain relief. 66.7% (8/12) of subjects with Grade 2 OA had ≥50% pain relief at 12 months. 74.1% (20/27) of subjects with Grade 3 OA had ≥50% pain relief at 12 months. 55.6% (15/27) of subjects with Grade 4 OA had ≥50% pain relief at 12 months. This data suggests that subjects with varying grades of OA may all receive benefit from CRFA treatment.

### Adverse events

AEs occurring during the first 6 months of this trial were previously reported [20]. There were 47 reported AEs in the 6–12-month period in subjects in the CRFA cohort. All of these AEs were deemed unrelated or unlikely related to the procedure. There were 8 reported AEs in the 6–12-month period in subjects in the HA cohort, all of which were deemed unrelated or unlikely relationship to procedure. There were 68 adverse events reported in the crossover cohort (Additional file 6 Table 6). Of these, 62 were unrelated to the procedure, 1 was unlikely related, 2 were possibly related and 3 were probably related to procedure. No events were deemed definitely related to procedure. All events reported in the 6–12-month follow-up period were similar to events reported in the 1–6-month follow-up period. AEs related to CRFA were similar to other clinical trials reporting 12-month outcomes [15]. No evidence of impaired proprioception or Charcot joints were identified during the study period. No SAEs related to either procedure were noted, and overall AE profiles were similar between all 3 cohorts (CRFA, HA and crossover).

## Discussion

There is currently a demand for prospective studies evaluating the methods and techniques used for nonoperative management of knee OA pain and disability [12, 28]. The number of patients experiencing symptoms associated with knee OA is increasing dramatically. While effective, TKR is not always necessary or indicated, and most patients benefit from nonoperative management of symptoms during disease progression. This study demonstrated that CRFA was effective at pain relief, reduction of stiffness, and improvement in physical function, global outcomes and quality of life at 12 months, and that patients who crossed over from HA demonstrated improvements in all the same domains.

Patients in the original CRFA cohort demonstrated pain relief that extended to the 12-month timepoint with a significant improvement from baseline. The percentage of subjects within the CRFA cohort in this trial reporting ≥50% pain relief at the 12-month timepoint (65.2%) was similar to what has been reported in previous CRFA trials (65.4%) [15]. Furthermore, the mean decrease in NRS of subjects in this CRFA cohort (4.0) matched with previously reported 12-month results of other trials (4.3) [15].

In addition to improvements in total WOMAC score, subjects within the CRFA cohort also experienced improvements in WOMAC pain score that were above the 12–18% improvement considered the minimal clinically important difference in OA [29]. Other randomized, blinded clinical trials conducted with HA have shown modest benefits in WOMAC scores. Lin et al. showed statistically significant improvements in WOMAC scores at 1 month (14% improvement) [30]. However, at the 6-month timepoint, there was a 0% improvement compared to baseline. At 12 months, there was a 6% decrease in WOMAC score, suggesting the durability of HA was attenuated over time.

CRFA was associated with an increase in the general health of subjects, as reported by self-reported measures GPE and EQ-5D-5 L. The majority (63.6%) of subjects receiving CRFA reported improved GPE at the 12-month timepoint. Those within the CRFA reported a change in EQ-5D-5 L of 0.14 points from baseline, which exceeds the minimal clinically important difference in EQ-5D-5 L of 0.074 [31].

This study also demonstrated that CRFA can be offered to patients who continue to experience pain and discomfort following viscosupplementation injections. The previously reported 6-month results from this trial indicate that HA does not provide extended pain relief when managing chronic knee pain caused by osteoarthritis [20]. Additionally, the majority (83%) of subjects within the HA cohort elected to crossover and receive CRFA after 6 months. Once these crossover subjects received CRFA, there was a significant reduction in pain relief after CRFA treatment. A higher percentage of subjects within this cohort (64.5%) reported ≥50% pain relief at the 12-month timepoint than in previous trials (48.6%) [15], although the crossover cohort in the Davis et al. trial first received IAS then CRFA. Other trials studying pain relief following HA have reported sustained decreases in WOMAC pain subscale score [32]. Subjects reported a baseline of 7.52 ± 0.58 that remained lower at 6 weeks (4.66 ± 0.47), 12 weeks (5.00 ± 0.60), 24 weeks (5.00 ± 0.50) and 52 weeks (4.00 ± 0.60).

Of note, patients who elected to crossover did experience measurable improvements in pain, as their NRS scores lowered 2.0 points at the 6-month time period following crossover to receive CRFA, representing an improvement of 36.1%. This change in NRS meets the minimal clinically important change previously reported as a decrease by 2.0 or a percent chance score of − 33.0% [33]. Additionally, during this time period, subjects within this cohort saw a 27.7% improvement in WOMAC pain score, which exceeds the minimal clinically important difference in osteoarthritis [29].

Quality of life measurements showed improvements in the crossover cohort. Subjects in this cohort reported an increase in GPE from their 6-month to 12-month timepoint. Previous studies have demonstrated that HA has an approximate 6-month durability of patient impression. Chevalier et al. showed that 33.9% of subjects reported doing ‘very well’ or ‘well’ at 26 weeks after treatment, as measured by patient global assessment [11], which closely mirror the GPE scores at the 6-month timepoint in this trial. However, GPE scores increased significantly in the crossover cohort following CRFA treatment, suggesting that the improvements in patient impression were the result of CRFA treatment. Furthermore, this cohort saw increases in EQ-5D-5 L score after receiving CRFA, suggesting that the overall impression of treatment was positive following the CRFA procedure.

Several subjects (*n* = 14) within the origin HA cohort that did not crossover to receive CRFA. These subjects were not deemed medically appropriate candidates for CRFA by their treating physician. Subjects that did not crossover to receive CRFA reporting 12-month outcomes (*n* = 11) saw long-term benefits in terms of pain and function at this timepoint. Study investigators were surprised to see long-term pain relief from this cohort, as summaries of clinical literature do not suggest long-term durability of this treatment. One clinical trial demonstrated that HA treatment had a durability of approximately 3 months [34] while other studies have demonstrated that pain relief can extend to 26 weeks [11]. Meta-analyses assessing the benefits and risks of viscosupplementation for adults with symptomatic knee OA have concluded that it is associated with a small and clinically irrelevant benefit [35]. However, the clinical trial reported herein was not powered to examine the long-term durability of HA and firm conclusions cannot be made on such a small sample size.

Overall, AE profiles were similar across cohorts and were consistent with published literature. No SAEs related to either procedure were observed. Skin burns have been reported following traditional RF procedures [36]. One instance of skin burn was previously reported following CRFA in this trial at the lower medial needle insertion site, which resolved on its own [20]. Review articles have suggested that vascular injuries may be a risk during CRFA procedures [37], and there are case reports of hemarthrosis [38] and septic arthrosis [39] following CRFA for knee OA but none have been observed in the clinical trials previously conducted [15—17,19,20], nor were observed in this study.

Since trial inception, there have been a number of anatomical studies of the knee published. Genicular nerve targets for this trial were based on available literature [22]. Tran et al demonstrated a total of 10 nerves innervating the knee [40]. Fonkue et al recently published work describing 5 potential genicular nerve targets [41]. It is possible that optimization of the procedure informed on recent anatomical work may lead to even better clinical outcomes in future studies. However, the targets used in this study resulted in consistent, favorable clinical outcomes with prior CRFA studies [15, 16, 19].

Limitations of this study included the lack of blinding as a result of pragmatic study design. The open-label nature of the trial allowed the opportunity for bias. Additionally, this trial was designed as a single-arm crossover study, so subjects receiving CRFA were not eligible to receive HA injections. Some subjects with minimal or no pain (i.e. an NRS score of 0.0) crossed over from HA to CRFA. However, the majority of the subjects within the crossover cohort did report significant improvements in pain following CRFA.

## Conclusions

Overall, these results closely mirrored the durability of CRFA demonstrated in previous trials extending 12 months [15—17]. Furthermore, this study demonstrated that subjects who received HA prior to CRFA can still receive substantial benefit from CRFA. At the 12-month timepoint, subjects in both CRFA and crossover cohorts reported lowered NRS pain scores. These study results suggest that patients may benefit by receiving CRFA initially rather than HA, but those that receive CRFA after HA may still expect improvement in outcomes.

## Supplementary information

**Additional file 1: Table 1.** Baseline Demographics.

**Additional file 2: Table 2.** NRS Through 12 Months by Actual Visits.

**Additional file 3: Table 3.** WOMAC Total Normalized Score Through 12 Months.

**Additional file 4: Table 4.** GPE Score Through 12 Months.

**Additional file 5: Table 5.** EQ-5D-5L Index Score Through 12 Months.

**Additional file 6: Table 6.** Distribution of All Adverse Events for All Subjects Treated with CRFA.

## Data Availability

The data that support the findings of this study are available from Avanos Medical, Inc. but restrictions apply to the availability of these data, which were used under license for the current study, and so are not publicly available. Data are however available from the authors upon reasonable request and with permission of Avanos Medical, Inc.

## Abbreviations

CRFA: Cooled radiofrequency ablation
HA: Hyaluronic acid
IAS: Intra-articular steroid
NSAIDs: Nonsteroidal anti-inflammatory drugs
OA: Osteoarthritis
TKA: Total knee arthroplasty
CT: Computed tomography
MRI: Magnetic resonance imaging
NRS: Numeric rating scale
IFU: Instructions for Use
