# Supplementary material for: Cooled radiofrequency ablation provides extended clinical utility in the management of knee osteoarthritis: 12-month results from a prospective, multi-center, randomized, cross-over trial comparing cooled radiofrequency ablation to a single hyaluronic acid injection
Source: BMC Musculoskelet Disord. 2020 Jun 9;21:363. doi: 10.1186/s12891-020-03380-5 (PMC7285532; doi:10.1186/s12891-020-03380-5)
Supplement: Supplementary file 1 — Additional file 1: Table 1. Baseline Demographics. [file 12891_2020_3380_MOESM1_ESM.docx]

| **Baseline Demographics** | | |  |
| --- | --- | --- | --- |
|  | **CRFA** | **Crossover** |  |
| **Number of Subjects** | **89** | **68** |  |
| **Age at Consent (years)** |  |  |  |
| N | 89 | 68 |  |
| Mean (SD) | 63.3 (10.7) | 62.8 (9.5) |  |
| Median | 63.7 | 61.5 |  |
| Min., Max. | 37.8, 84.3 | 45.0, 90.8 |  |
| P-value (difference between groups) | 0.7640* | |  |
| **Gender (n/N (%))** |  |  |  |
| Female | 52/89 (58.4) | 40/68 (58.8) |  |
| Male | 37/89 (41.6) | 28/68 (41.2) |  |
| P-value (difference between groups) | 0.9601^††^ | |  |
| **Race (n/N (%), not mutually exclusive)** |  |  |  |
| White | 83/89 (93.3) | 63/68 (92.6) |  |
| Black or African American | 3/89 (3.4) | 5/68 (7.4) |  |
| Asian | 2/89 (2.2) | 0/68 (0.0) |  |
| Native Hawaiian or Other Pacific Islander | 0/89 (0.0) | 0/68 (0.0) |  |
| American Indian or Alaska Native | 0/89 (0.0) | 0/68 (0.0) |  |
| Other | 1/89 (1.1) | 0/68 (0.0) |  |
| P-value (difference between groups, White vs. Not White) | 1.0000 |  |  |
| **Ethnicity (n/N (%))** |  |  |  |
| Hispanic or Latino | 0/89 (0.0) | 1/68 (1.5) |  |
| Not Hispanic or Latino | 89/89 (100.0) | 67/68 (98.5) |  |
| P-value (difference between groups) | 0.4331 | |  |
| **Height (in)** |  |  |  |
| N | 89 | 68 |  |
| Mean (SD) | 67.3 (4.4) | 67.1 (4.0) |  |
| Median | 67.5 | 66.0 |  |
| Min., Max. | 56.0, 78.0 | 59.0, 75.0 |  |
| P-value (difference between groups) | 0. 7396* | |  |
| **Weight (lbs)** |  |  |  |
| N | 89 | 68 |  |
| Mean (SD) | 208.9 (46.1) | 196.1 (34.3) |  |
| Median | 203.0 | 199.8 |  |
| Min., Max. | 115.0, 338.5 | 98.0, 270.0 |  |
| P-value (difference between groups) | 0.0485* | |  |
| **BMI (kg/ m^2^)** |  |  |  |
| N | 89 | 68 |  |
| Mean (SD) | 32.2 (5.2) | 30.6 (4.7) |  |
| Median | 32.5 | 30.0 |  |
| Min., Max. | 20.4, 41.3 | 18.8, 39.9 |  |
| P-value (difference between groups) | 0.0459* | |  |
| CRFA, cooled radiofrequency ablation; N, total number of patients; SD, standard deviation; Min, minimum; Max, maximum; n, number of patients to which description is applicable; OA, osteoarthritis; CI, confidence interval; NRS, Numeric Rating Scale; *t-test for two independent means; ^††^chi-squared test for proportions; ^†^Fisher exact test for proportions;**Wilcoxon/Wilcoxon-Mann-Whitney test for location; ^Wilcoxon rank sum test for two independent samples. | | |  |
