# Supplementary material for: Cooled radiofrequency ablation provides extended clinical utility in the management of knee osteoarthritis: 12-month results from a prospective, multi-center, randomized, cross-over trial comparing cooled radiofrequency ablation to a single hyaluronic acid injection
Source: BMC Musculoskelet Disord. 2020 Jun 9;21:363. doi: 10.1186/s12891-020-03380-5 (PMC7285532; doi:10.1186/s12891-020-03380-5)
Supplement: Supplementary file 2 — Additional file 2: Table 2. NRS Through 12 Months by Actual Visits. [file 12891_2020_3380_MOESM2_ESM.docx]

Appendix Table 2. NRS Through 12 Months by Actual Visits

|  | | | | | | | | | | |
| --- | --- | --- | --- | --- | --- | --- | --- | --- | --- | --- |
|  | **Baseline** | | **1 Month** | | **3 Month** | | **6 Month** | | **12 Month** | |
|  | **CRFA** | **XO** | **CRFA** | **XO** | **CRFA** | **XO** | **CRFA** | **XO** | **CRFA** | **XO** |
| **USUAL Level of Pain** |  |  |  |  |  |  |  |  |  |  |
| N | 89 | 68 | 86 | 67 | 84 | 67 | 76 | 68 | 66 | 62 |
| Mean | 6.9 | 7.0 | 3.0 | 4.2 | 2.3 | 4.4 | 2.7 | 5.0 | 2.8 | 3.0 |
| SD | 0.8 | 1.0 | 2.4 | 2.4 | 2.1 | 2.3 | 2.3 | 2.5 | 2.4 | 2.4 |
| Median | 7.0 | 7.0 | 3.0 | 4.0 | 2.0 | 4.0 | 2.0 | 5.0 | 2.5 | 3.0 |
| Minimum | 6.0 | 6.0 | 0.0 | 0.0 | 0.0 | 0.0 | 0.0 | 0.0 | 0.0 | 0.0 |
| Maximum | 9.0 | 10.0 | 10.0 | 10.0 | 9.0 | 10.0 | 10.0 | 10.0 | 9.0 | 9.0 |
| 95% CI for the mean | (6.7, 7.1) | (6.8, 7.3) | (2.5, 3.5) | (3.6, 4.8) | (1.8, 2.7) | (3.8, 4.9) | (2.2, 3.2) | (4.4, 5.6) | (2.2, 3.4) | (2.4, 3.6) |
| Difference between means (CRFA-HA) and 95% CI | -0.2 (-0.4, 0.1) | | -1.2 (-2.0, -0.5) | | -2.1 (-2.8, -1.4) | | -2.3 (-3.1, -1.5) | | -0.2 (-1.1, 0.6) | |
| P-value (difference between groups) | 0.2862* | | 0.0018* | | <0.0001* | | <0.0001* | | 0.6184* | |
| **Change from Baseline in USUAL Level of Pain** |  |  |  |  |  |  |  |  |  |  |
| N | -- | -- | 86 | 67 | 84 | 67 | 76 | 68 | 66 | 62 |
| Mean | -- | -- | 3.9 | 2.8 | 4.6 | 2.7 | 4.1 | 2.0 | 4.1 | 4.0 |
| SD | -- | -- | 2.4 | 2.2 | 2.1 | 2.2 | 2.2 | 2.3 | 2.4 | 2.6 |
| Median | -- | -- | 4.5 | 3.0 | 5.0 | 3.0 | 4.5 | 2.0 | 4.0 | 4.0 |
| Minimum | -- | -- | -3.0 | -2.0 | -2.0 | -2.0 | -1.0 | -2.0 | -2.0 | -2.0 |
| Maximum | -- | -- | 8.0 | 8.0 | 9.0 | 7.0 | 8.0 | 7.0 | 8.0 | 10.0 |
| 95% CI for the mean | -- | -- | (3.4, 4.4) | (2.3, 3.4) | (4.1, 5.0) | (2.1, 3.2) | (3.6, 4.7) | (1.5, 2.6) | (3.5, 4.6) | (3.3, 4.7) |
| Difference between means (CRFA-HA) and 95% CI | -- | -- | 1.1 (0.3, 1.8) | | 1.9 (1.2, 2.6) | | 2.1 (1.4, 2.9) | | 0.1 (-0.8, 0.9) | |
| P-value (difference between groups) | -- | -- | 0.0052* | | <0.0001* | | <0.0001* | | 0.8901* | |
| P-value (change from Baseline) | -- | -- | <0.0001^$^ | <0.0001^$^ | <0.0001^$^ | <0.0001^$^ | <0.0001^$^ | <0.0001^$^ | <0.0001^$^ | <0.0001^$^ |
| **Percent Change from Baseline in USUAL Level of Pain** |  |  |  |  |  |  |  |  |  |  |
| N | -- | -- | 86 | 67 | 84 | 67 | 76 | 68 | 66 | 62 |
| Mean | -- | -- | 56.8 | 40.9 | 67.1 | 38.2 | 60.9 | 29.4 | 59.3 | 56.6 |
| SD | -- | -- | 34.3 | 31.1 | 30.4 | 31.3 | 32.1 | 33.1 | 34.4 | 34.5 |
| Median | -- | -- | 62.5 | 42.9 | 75.0 | 37.5 | 66.7 | 26.8 | 61.9 | 59.8 |
| Minimum | -- | -- | -42.9 | -33.3 | -28.6 | -28.6 | -16.7 | -33.3 | -33.3 | -33.3 |
| Maximum | -- | -- | 100.0 | 100.0 | 100.0 | 100.0 | 100.0 | 100.0 | 100.0 | 100.0 |
| 95% CI for the mean | -- | -- | (49.4, 64.1) | (33.3, 48.5) | (60.5, 73.7) | (30.5, 45.8) | (53.5, 68.2) | (21.4, 37.4) | (50.8, 67.7) | (47.9, 65.4) |
| Difference between means (CRFA-HA) and 95% CI | -- | -- | 15.9 (5.3, 26.5) | | 28.9 (18.9, 38.9) | | 31.5 (20.7, 42.2) | | 2.7 (-9.4, 14.7) | |
| P-value (difference between groups) | -- | -- | 0.0036* | | <0.0001* | | <0.0001* | | 0.6631* | |
| **Subjects with ≥50% Improvement from Baseline (n/N (%))** |  |  |  |  |  |  |  |  |  |  |
| Yes | -- | -- | 56/86 (65.1) | 26/67 (38.8) | 64/84 (76.2) | 29/67 (43.3) | 54/76 (71.1) | 20/68 (29.4) | 43/66 (65.2) | 40/62 (64.5) |
| No | -- | -- | 30/86 (34.9) | 41/67 (61.2) | 20/84 (23.8) | 38/67 (56.7) | 22/76 (28.9) | 48/68 (70.6) | 23/66 (34.8) | 22/62 (35.5) |
| P-value (difference between groups) | -- |  | 0.0012^††^ | | <0.0001^††^ | | <0.0001^††^ | | 0.9400^††^ | |
| **T-test for two independent means, **Wilcoxon rank sum test for two independent samples, ^$^paired t-test, ^†^Fisher exact test for two categorical variables, ^††^Chi-square test for two categorical variables  Program: HYH12 output Numeric Rating Scale by Visits.sas Data Source: hyh12_nrs Date Run: 19NOV2019 - 15:46*  (CRFA = cooled radiofrequency ablation, XO = crossover) | | | | | | | | | | |
