# Supplementary material for: Cooled radiofrequency ablation provides extended clinical utility in the management of knee osteoarthritis: 12-month results from a prospective, multi-center, randomized, cross-over trial comparing cooled radiofrequency ablation to a single hyaluronic acid injection
Source: BMC Musculoskelet Disord. 2020 Jun 9;21:363. doi: 10.1186/s12891-020-03380-5 (PMC7285532; doi:10.1186/s12891-020-03380-5)
Supplement: Supplementary file 3 — Additional file 3: Table 3. WOMAC Total Normalized Score Through 12 Months. [file 12891_2020_3380_MOESM3_ESM.docx]

Appendix Table 3. WOMAC Total Normalized Score Through 12 Months

|  | | | | | | | | | | |
| --- | --- | --- | --- | --- | --- | --- | --- | --- | --- | --- |
|  | **Baseline** | | **1 Month** | | **3 Month** | | **6 Month** | | **12 Month** | |
|  | **CRFA** | **XO** | **CRFA** | **XO** | **CRFA** | **XO** | **CRFA** | **XO** | **CRFA** | **XO** |
| **WOMAC Total Score** |  |  |  |  |  |  |  |  |  |  |
| N | 88 | 68 | 87 | 67 | 84 | 67 | 76 | 68 | 66 | 62 |
| Mean | 66.1 | 68.8 | 36.6 | 46.7 | 32.2 | 51.2 | 33.6 | 58.1 | 33.2 | 38.4 |
| SD | 13.2 | 11.8 | 23.1 | 20.3 | 23.1 | 19.1 | 22.9 | 19.4 | 23.2 | 22.3 |
| Median | 65.6 | 67.7 | 31.3 | 49.0 | 29.2 | 51.0 | 31.8 | 61.5 | 31.8 | 39.1 |
| Minimum | 28.1 | 38.5 | 0.0 | 7.3 | 0.0 | 11.5 | 0.0 | 6.3 | 0.0 | 0.0 |
| Maximum | 92.7 | 97.9 | 100.0 | 92.7 | 79.2 | 92.7 | 89.6 | 96.9 | 87.5 | 84.4 |
| 95% CI for the mean | (63.3, 68.9) | (66.0, 71.7) | (31.7, 41.5) | (41.8, 51.7) | (27.1, 37.2) | (46.6, 55.9) | (28.4, 38.9) | (53.4, 62.8) | (27.5, 38.9) | (32.7, 44.1) |
| Difference between means (CRFA-HA) and 95% CI | -2.8 (-6.8, 1.3) | | -10.1 (-17.2, -3.1) | | -19.1 (-26.0, -12.1) | | -24.4 (-31.5, -17.4) | | -5.2 (-13.2, 2.8) | |
| P-value (difference between groups) | 0.1800* | | 0.0052* | | <0.0001* | | <0.0001* | | 0.1996* | |
| **Improvement from Baseline in WOMAC Total Score** |  |  |  |  |  |  |  |  |  |  |
| N | -- | -- | 86 | 67 | 83 | 67 | 75 | 68 | 65 | 62 |
| Mean | -- | -- | 29.4 | 22.0 | 33.1 | 17.5 | 31.5 | 10.7 | 30.7 | 30.1 |
| SD | -- | -- | 22.5 | 18.1 | 24.4 | 17.8 | 23.0 | 16.1 | 25.2 | 19.3 |
| Median | -- | -- | 30.2 | 20.8 | 31.3 | 15.6 | 29.6 | 8.0 | 30.2 | 28.1 |
| Minimum | -- | -- | -43.8 | -10.4 | -22.9 | -17.7 | -10.4 | -25.0 | -30.2 | -10.4 |
| Maximum | -- | -- | 84.4 | 61.5 | 84.4 | 62.5 | 84.4 | 67.7 | 88.5 | 72.9 |
| 95% CI for the mean | -- | -- | (24.5, 34.2) | (17.6, 26.5) | (27.8, 38.4) | (13.2, 21.9) | (26.2, 36.8) | (6.8, 14.6) | (24.5, 37.0) | (25.2, 35.0) |
| Difference between means (CRFA-HA) and 95% CI | -- | -- | 7.3 (0.7, 14.0) | | 15.6 (8.8, 22.4) | | 20.8 (14.3, 27.3) | | 0.6 (-7.3, 8.5) | |
| P-value (difference between groups) | -- | -- | 0.0314* | | <0.0001* | | <0.0001* | | 0.8820* | |
| P-value (change from Baseline) | -- | -- | <0.0001^$^ | <0.0001^$^ | <0.0001^$^ | <0.0001^$^ | <0.0001^$^ | <0.0001^$^ | <0.0001^$^ | -- |
| **Percent Improvement from Baseline in WOMAC Total Score** |  |  |  |  |  |  |  |  |  |  |
| N | -- | -- | 86 | 67 | 83 | 67 | 75 | 68 | 65 | 62 |
| Mean | -- | -- | 44.7 | 32.1 | 49.9 | 25.2 | 48.2 | 15.9 | 46.2 | 45.1 |
| SD | -- | -- | 33.2 | 26.9 | 36.3 | 25.9 | 32.3 | 24.6 | 38.1 | 29.8 |
| Median | -- | -- | 49.3 | 29.7 | 56.3 | 26.3 | 45.2 | 10.7 | 48.5 | 41.5 |
| Minimum | -- | -- | -77.8 | -20.4 | -40.7 | -34.7 | -13.7 | -40.0 | -63.0 | -16.4 |
| Maximum | -- | -- | 100.0 | 88.5 | 100.0 | 84.5 | 100.0 | 87.5 | 100.0 | 100.0 |
| 95% CI for the mean | -- | -- | (37.5, 51.8) | (25.6, 38.7) | (41.9, 57.8) | (18.8, 31.5) | (40.8, 55.6) | (10.0, 21.9) | (36.8, 55.7) | (37.5, 52.6) |
| Difference between means (CRFA-HA) and 95% CI | -- | -- | 12.5 (2.7, 22.4) | | 24.7 (14.6, 34.8) | | 32.3 (22.8, 41.7) | | 1.2 (-10.9, 13.2) | |
| P-value (difference between groups) | -- | -- | 0.0131* | | <0.0001* | | <0.0001* | | 0.8491* | |
| **T-test for two independent means, **Wilcoxon rank sum test for two independent samples, ^$^paired t-test, ^†^Fisher exact test for two categorical variables, ^††^Chi-square test for two categorical variables  Program: HYH12 output WOMAC Norm by XO.sas Data Source: hyh12_womac Date Run: 29AUG2019 - 21:15* | | | | | | | | | | |

(CRFA = cooled radiofrequency ablation, XO = crossover)
