# Supplementary material for: Cooled radiofrequency ablation provides extended clinical utility in the management of knee osteoarthritis: 12-month results from a prospective, multi-center, randomized, cross-over trial comparing cooled radiofrequency ablation to a single hyaluronic acid injection
Source: BMC Musculoskelet Disord. 2020 Jun 9;21:363. doi: 10.1186/s12891-020-03380-5 (PMC7285532; doi:10.1186/s12891-020-03380-5)
Supplement: Supplementary file 4 — Additional file 4: Table 4. GPE Score Through 12 Months. [file 12891_2020_3380_MOESM4_ESM.docx]

Appendix Table 4. GPE Score Through 12 Months

|  | | | | | | | | |
| --- | --- | --- | --- | --- | --- | --- | --- | --- |
|  | **1 Month** | | **3 Month** | | **6 Month** | | **12 Month** | |
| **n/N (%)** | **CRFA** | **XO** | **CRFA** | **XO** | **CRFA** | **XO** | **CRFA** | **XO** |
| **Global Perceived Effect Score** |  |  |  |  |  |  |  |  |
| 1-Worst ever | 0/87 (0.0) | 0/67 (0.0) | 1/84 (1.2) | 0/67 (0.0) | 1/76 (1.3) | 1/68 (1.5) | 0/66 (0.0) | 0/62 (0.0) |
| 2-Much worse | 1/87 (1.1) | 1/67 (1.5) | 0/84 (0.0) | 1/67 (1.5) | 0/76 (0.0) | 2/68 (2.9) | 2/66 (3.0) | 1/62 (1.6) |
| 3-Worse | 5/87 (5.7) | 4/67 (6.0) | 4/84 (4.8) | 14/67 (20.9) | 2/76 (2.6) | 23/68 (33.8) | 4/66 (6.1) | 4/62 (6.5) |
| 4-Not improved but not worse | 12/87 (13.8) | 21/67 (31.3) | 13/84 (15.5) | 21/67 (31.3) | 18/76 (23.7) | 20/68 (29.4) | 18/66 (27.3) | 18/62 (29.0) |
| 5-Improved | 27/87 (31.0) | 25/67 (37.3) | 21/84 (25.0) | 21/67 (31.3) | 24/76 (31.6) | 13/68 (19.1) | 11/66 (16.7) | 13/62 (21.0) |
| 6-Much Improved | 34/87 (39.1) | 16/67 (23.9) | 37/84 (44.0) | 10/67 (14.9) | 25/76 (32.9) | 8/68 (11.8) | 26/66 (39.4) | 20/62 (32.3) |
| 7-Best Ever | 8/87 (9.2) | 0/67 (0.0) | 8/84 (9.5) | 0/67 (0.0) | 6/76 (7.9) | 1/68 (1.5) | 5/66 (7.6) | 6/62 (9.7) |
| P-value (difference between groups) | 0.0010** | | <0.0001** | | <0.0001** | | 0.8661** | |
| **Distribution of Global Perceived Effect Score** |  |  |  |  |  |  |  |  |
| Not Improved/Worse | 18/87 (20.7) | 26/67 (38.8) | 18/84 (21.4) | 36/67 (53.7) | 21/76 (27.6) | 46/68 (67.6) | 24/66 (36.4) | 23/62 (37.1) |
| Improved | 69/87 (79.3) | 41/67 (61.2) | 66/84 (78.6) | 31/67 (46.3) | 55/76 (72.4) | 22/68 (32.4) | 42/66 (63.6) | 39/62 (62.9) |
| P-value (difference between groups) | 0.0136^††^ | | <0.0001^††^ | | <0.0001^††^ | | 0.9315^††^ | |
| ***Wilcoxon-Mann-Whitney test for location, ^†^Fisher exact test for two categorical variables, ^††^Chi-square test for two categorical variables  Program: HYH12 output Global Perceived Effect by XO.sas Data Source: hyh12_gpe Date Run: 29AUG2019 - 21:34* | | | | | | | | |

(CRFA = cooled radiofrequency ablation, XO = crossover)
