# Supplementary material for: Cooled radiofrequency ablation provides extended clinical utility in the management of knee osteoarthritis: 12-month results from a prospective, multi-center, randomized, cross-over trial comparing cooled radiofrequency ablation to a single hyaluronic acid injection
Source: BMC Musculoskelet Disord. 2020 Jun 9;21:363. doi: 10.1186/s12891-020-03380-5 (PMC7285532; doi:10.1186/s12891-020-03380-5)
Supplement: Supplementary file 5 — Additional file 5: Table 5. EQ-5D-5L Index Score Through 12 Months. [file 12891_2020_3380_MOESM5_ESM.docx]

Appendix Table 5. EQ-5D-5L Index Score Through 12 Months

|  | | | | | | | | | | |
| --- | --- | --- | --- | --- | --- | --- | --- | --- | --- | --- |
|  | **Baseline** | | **1 Month** | | **3 Month** | | **6 Month** | | **12 Month** | |
|  | **CRFA** | **XO** | **CRFA** | **XO** | **CRFA** | **XO** | **CRFA** | **XO** | **CRFA** | **XO** |
| **EQ-5D-5L Index Score** |  |  |  |  |  |  |  |  |  |  |
| N | 88 | 68 | 87 | 67 | 83 | 67 | 76 | 68 | 66 | 62 |
| Mean | 0.67 | 0.65 | 0.79 | 0.75 | 0.82 | 0.74 | 0.80 | 0.70 | 0.81 | 0.79 |
| SD | 0.12 | 0.13 | 0.10 | 0.11 | 0.10 | 0.10 | 0.11 | 0.11 | 0.10 | 0.14 |
| Median | 0.70 | 0.65 | 0.80 | 0.79 | 0.80 | 0.78 | 0.80 | 0.71 | 0.80 | 0.80 |
| Minimum | 0.29 | 0.34 | 0.50 | 0.43 | 0.56 | 0.46 | 0.43 | 0.43 | 0.58 | 0.35 |
| Maximum | 0.83 | 0.83 | 1.00 | 1.00 | 1.00 | 1.00 | 1.00 | 1.00 | 1.00 | 1.00 |
| 95% CI for the mean | (0.65, 0.70) | (0.62, 0.68) | (0.77, 0.81) | (0.73, 0.78) | (0.80, 0.84) | (0.72, 0.76) | (0.78, 0.83) | (0.67, 0.73) | (0.78, 0.83) | (0.75, 0.82) |
| Difference between means (CRFA-HA) and 95% CI | 0.02 (-0.02, 0.06) | | 0.04 (0.01, 0.07) | | 0.08 (0.05, 0.11) | | 0.10 (0.07, 0.14) | | 0.02 (-0.02, 0.06) | |
| P-value (difference between groups) | 0.2545* | | 0.0248* | | <0.0001* | | <0.0001* | | 0.3048* | |
| **Change from Baseline in EQ-5D-5L Index Score** |  |  |  |  |  |  |  |  |  |  |
| N | -- | -- | 86 | 67 | 82 | 67 | 75 | 68 | 65 | 62 |
| Mean | -- | -- | 0.12 | 0.10 | 0.14 | 0.09 | 0.12 | 0.05 | 0.12 | 0.13 |
| SD | -- | -- | 0.14 | 0.12 | 0.13 | 0.13 | 0.14 | 0.14 | 0.14 | 0.15 |
| Median | -- | -- | 0.09 | 0.08 | 0.15 | 0.06 | 0.09 | 0.04 | 0.08 | 0.16 |
| Minimum | -- | -- | -0.21 | -0.08 | -0.23 | -0.23 | -0.17 | -0.24 | -0.17 | -0.35 |
| Maximum | -- | -- | 0.56 | 0.45 | 0.42 | 0.45 | 0.56 | 0.45 | 0.56 | 0.45 |
| 95% CI for the mean | -- | -- | (0.09, 0.15) | (0.07, 0.13) | (0.11, 0.17) | (0.06, 0.12) | (0.09, 0.16) | (0.01, 0.08) | (0.09, 0.16) | (0.09, 0.17) |
| Difference between means (CRFA-HA) and 95% CI | -- | -- | 0.01 (-0.03, 0.06) | | 0.05 (0.01, 0.09) | | 0.08 (0.03, 0.12) | | -0.01 (-0.06, 0.04) | |
| P-value (difference between groups) | -- | -- | 0.5525* | | 0.0154* | | 0.0013* | | 0.7558* | |
| P-value (change from Baseline) | -- | -- | <0.0001^$^ | <0.0001^$^ | <0.0001^$^ | <0.0001^$^ | <0.0001^$^ | 0.0053^$^ | <0.0001^$^ | <0.0001^$^ |
| **Percent Change from Baseline in EQ-5D-5L Index Score** |  |  |  |  |  |  |  |  |  |  |
| N | -- | -- | 86 | 67 | 82 | 67 | 75 | 68 | 65 | 62 |
| Mean | -- | -- | 21.32 | 19.81 | 24.34 | 18.15 | 21.37 | 11.12 | 21.08 | 24.08 |
| SD | -- | -- | 27.53 | 27.47 | 24.74 | 27.35 | 26.38 | 28.05 | 25.43 | 29.58 |
| Median | -- | -- | 12.71 | 12.23 | 20.91 | 9.82 | 12.46 | 5.36 | 11.45 | 22.35 |
| Minimum | -- | -- | -29.97 | -13.49 | -29.21 | -28.48 | -26.51 | -33.48 | -21.39 | -48.22 |
| Maximum | -- | -- | 127.27 | 131.56 | 95.68 | 131.56 | 127.27 | 133.04 | 127.27 | 133.04 |
| 95% CI for the mean | -- | -- | (15.42, 27.22) | (13.12, 26.51) | (18.90, 29.77) | (11.47, 24.82) | (15.30, 27.43) | (4.33, 17.91) | (14.78, 27.38) | (16.57, 31.59) |
| Difference between means (CRFA-HA) and 95% CI | -- | -- | 1.50 (-7.35, 10.36) | | 6.19 (-2.25, 14.64) | | 10.25 (1.25, 19.25) | | -3.00 (-12.67, 6.68 | |
| P-value (difference between groups) | -- | -- | 0.7375* | | 0.1494* | | 0.0259* | | 0.5409* | |
| **T-test for two independent means, **Wilcoxon rank sum test for two independent samples, ^$^paired t-test  Program: HYH12 output EQ-5D-5L Index by Visits.sas Data Source: hyh12_eq5d5l Date Run: 22NOV2019 - 15:02* | | | | | | | | | | |

(CRFA = cooled radiofrequency ablation, XO = crossover)
