# Supplementary material for: Cooled radiofrequency ablation provides extended clinical utility in the management of knee osteoarthritis: 12-month results from a prospective, multi-center, randomized, cross-over trial comparing cooled radiofrequency ablation to a single hyaluronic acid injection
Source: BMC Musculoskelet Disord. 2020 Jun 9;21:363. doi: 10.1186/s12891-020-03380-5 (PMC7285532; doi:10.1186/s12891-020-03380-5)
Supplement: Supplementary file 6 — Additional file 6: Table 6. Distribution of All Adverse Events for All Subjects Treated with CRFA. [file 12891_2020_3380_MOESM6_ESM.docx]

Appendix Table 6. Distribution of All Adverse Events for All Subjects Treated with CRFA

|  | | | | | | | | | | |
| --- | --- | --- | --- | --- | --- | --- | --- | --- | --- | --- |
|  | **Total CRFA 0-6 Months^1^ N = 156 subjects** | | **XO 0-6 Months^2^ N = 68 subjects** | | **CRFA 0-6 Months^3^ N = 88 subjects** | | **CRFA 6-12 Months^4^ N = 76 subjects** | | **Total CRFA 0-12 Months^1^ N = 156 subjects** | |
|  | **Events N** | **Subjects n (%)** | **Events N** | **Subjects n (%)** | **Events N** | **Subjects n (%)** | **Events N** | **Subjects n (%)** | **Events N** | **Subjects n (%)** |
| **All Adverse Events** | **162** | **86 (55.1)** | **68** | **38 (55.9)** | **94** | **48 (54.5)** | **47** | **30 (39.5)** | **209** | **102 (65.4)** |
| Blood/Lymphatic | 2 | 2 (1.3) | 1 | 1 (1.5) | 1 | 1 (1.1) | 1 | 1 (1.3) | 3 | 3 (1.9) |
| Infection | 2 | 2 (1.3) | 1 | 1 (1.5) | 1 | 1 (1.1) | 0 | 0 (0.0) | 2 | 2 (1.3) |
| Not specified | 0 | 0 (0.0) | 0 | 0 (0.0) | 0 | 0 (0.0) | 1 | 1 (1.3) | 1 | 1 (1.3) |
| Cardiovascular | 3 | 3 (1.9) | 0 | 0 (0.0) | 3 | 3 (3.4) | 3 | 3 (3.9) | 6 | 6 (3.8) |
| Endocrine/Metabolic | 3 | 3 (1.9) | 1 | 1 (1.5) | 2 | 2 (2.3) | 1 | 1 (1.3) | 4 | 4 (2.6) |
| Extremities | 0 | 0 (0.0) | 0 | 0 (0.0) | 0 | 0 (0.0) | 1 | 1 (1.3) | 1 | 1 (.6) |
| Non-Knee | 0 | 0 (0.0) | 0 | 0 (0.0) | 0 | 0 (0.0) | 1 | 1 (1.3) | 1 | 1 (.6) |
| New Injury | 0 | 0 (0.0) | 0 | 0 (0.0) | 0 | 0 (0.0) | 1 | 1 (1.3) | 1 | 1 (.6) |
| Gastrointestinal | 2 | 1 (0.6) | 0 | 0 (0.0) | 2 | 1 (1.1) | 1 | 1 (1.3) | 3 | 2 (1.3) |
| Genitourinary | 1 | 1 (0.6) | 0 | 0 (0.0) | 1 | 1 (1.1) | 0 | 0 (0.0) | 1 | 1 (.6) |
| H.E.E.N.T | 2 | 2 (1.3) | 1 | 1 (1.5) | 1 | 1 (1.1) | 2 | 2 (2.6) | 4 | 4 (2.6) |
| Musculoskeletal | 118 | 72 (46.2) | 51 | 30 (44.1) | 67 | 42 (47.7) | 31 | 22 (28.9) | 149 | 87 (55.8) |
| Procedure | 5 | 4 (2.6) | 3 | 2 (2.9) | 2 | 2 (2.3) | 5 | 5 (6.6) | 10 | 9 (5.8) |
| Fall | 2 | 2 (1.3) | 2 | 2 (2.9) | 0 | 0 (0.0) | 1 | 1 (1.3) | 3 | 3 (1.9) |
| Pain | 50 | 42 (26.9) | 24 | 17 (25.0) | 26 | 25 (28.4) | 21 | 13 (17.1) | 71 | 52 (33.3) |
| Post-Procedure Pain | 13 | 11 (7.1) | 4 | 4 (5.9) | 9 | 7 (8.0) | 0 | 0 (0.0) | 13 | 11 (7.1) |
| New Injury | 19 | 14 (9.0) | 5 | 4 (5.9) | 14 | 10 (11.4) | 1 | 1 (1.3) | 20 | 15 (9.6) |
| Pes Bursa | 4 | 3 (1.9) | 0 | 0 (0.0) | 4 | 3 (3.4) | 0 | 0 (0.0) | 4 | 3 (1.9) |
| Numbness | 5 | 5 (3.2) | 2 | 2 (2.9) | 3 | 3 (3.4) | 0 | 0 (0.0) | 5 | 5 (3.2) |
| Instability | 4 | 4 (2.6) | 1 | 1 (1.5) | 3 | 3 (3.4) | 0 | 0 (0.0) | 4 | 4 (2.6) |
| Stiffness/Tightness | 5 | 4 (2.6) | 2 | 2 (2.9) | 3 | 2 (2.3) | 1 | 1 (1.3) | 6 | 5 (3.2) |
| Bruising/Swelling | 5 | 5 (3.2) | 4 | 4 (5.9) | 1 | 1 (1.1) | 1 | 1 (1.3) | 6 | 6 (3.8) |
| Other | 6 | 5 (3.2) | 4 | 3 (4.4) | 2 | 2 (2.3) | 1 | 1 (1.3) | 7 | 6 (3.8) |
| Neurological | 6 | 6 (3.8) | 3 | 3 (4.4) | 3 | 3 (3.4) | 3 | 3 (3.9) | 9 | 9 (5.8) |
| Respiratory | 0 | 0 (0.0) | 0 | 0 (0.0) | 0 | 0 (0.0) | 1 | 1 (1.3) | 1 | 1 (.6) |
| Skin | 3 | 3 (1.9) | 1 | 1 (1.5) | 2 | 2 (2.3) | 0 | 0 (0.0) | 3 | 3 (1.9) |
| Other | 22 | 18 (11.5) | 10 | 9 (13.2) | 12 | 9 (10.2) | 3 | 3 (3.9) | 25 | 21 (13.5) |
| Procedure | 1 | 1 (0.6) | 0 | 0 (0.0) | 1 | 1 (1.1) | 1 | 1 (1.3) | 2 | 2 (1.3) |
| Fall | 16 | 14 (9.0) | 7 | 6 (8.8) | 9 | 8 (9.1) | 2 | 2 (2.6) | 18 | 16 (10.3) |
| Pain | 1 | 1 (0.6) | 1 | 1 (1.5) | 0 | 0 (0.0) | 0 | 0 (0.0) | 1 | 1 (0.6) |
| Other | 4 | 4 (2.6) | 2 | 2 (2.9) | 2 | 2 (2.3) | 0 | 0 (0.0) | 4 | 4 (2.6) |
| *^1^Total CRFA group includes subjects who were either randomized to and treated with Cooled Radiofrequency Ablation or were randomized Hyaluronic Acid injection group, chose to cross over to Cooled Radiofrequency Ablation and were treated with CRFA.  ^2^Cross-Over group for 0-6 Months includes subjects who were randomized to Intra-Articular Steroid group, chose to cross over to Cooled Radiofrequency Ablation and were treated with CRFA.  ^3^CRFA group for 0-6 Months includes subjects who were randomized to and treated with Cooled Radiofrequency Ablation. ^4^CRFA group for 6-12 Months includes subjects who were randomized to and treated with Cooled Radiofrequency Ablation and completed the 6-Month visit.  Program: N/A Data Source: hyh12_adverse, hyh12_roster Date Run: 11SEP2019 - 21:31* | | | | | | | | | | |

(CRFA = cooled radiofrequency ablation, XO = crossover)
